# 665. Modified Superposition *in vitro* Susceptibility of Aztreonam and Ceftazidime/Avibactam for an NDM-5 Metallo-β-Lactamase-Producing *Klebsiella pneumoniae* Isolate

**DOI:** 10.1093/ofid/ofac492.717

**Published:** 2022-12-15

**Authors:** Justin Schmetterer, Kelley Merrick, Antonio Gallegos, Taylor Babiarz, Yao Lin

**Affiliations:** Presbyterian Healthcare Services, Albuquerque, New Mexico; Presbyterian Healthcare Services, Albuquerque, New Mexico; TriCore Reference Laboratories, Albuquerque, New Mexico; Midland Memorial Hospital, Midland, Texas; Abbvie, Irvine, California

## Abstract

**Background:**

We describe a novel *in vitro* susceptibility testing approach of aztreonam and ceftazidime/avibactam using a superposition method paired with confirmatory testing and molecular sequencing of a New Delhi metallo-β-lactamase (NDM)-5-producing *Klebsiella pneumoniae*.

**Methods:**

A modified strip superposition method with Epsilometer test (Etest) strips and Kirby Bauer disks was utilized to test the *in vitro* activity of aztreonam and ceftazidime/avibactam against the NDM-producing *K. pneumoniae*. The isolate was also sent for whole genome sequencing and tested for the *in vitro* activity of aztreonam and ceftazidime in combination with avibactam using a broth microdilution checkerboard assay.

Aztreonam and ceftazidime/avibactam in vitro testing with modified superposition method

**Figure:**
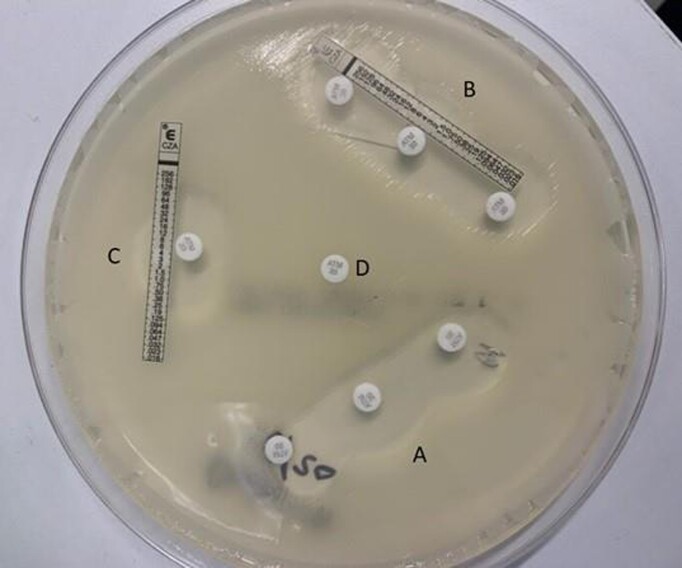

**Results:**

All zones of inhibition from the Etest superposition method were measured to be 22 mm in diameter (Clinical and Laboratory Standards Institute [CLSI] zone of inhibition for aztreonam susceptibility is ≥ 21 mm for Enterobacterales). The results of whole genome sequencing showed the *K. pneumoniae* isolate carried a metallo-β-lactamase (*bla*NDM-5), extended-spectrum β-lactamase (*bla*CTX-M-15), and other β-lactamases (*bla*SHV-11, *bla*TEM-1). Through the broth microdilution checkerboard method, the isolate demonstrated inhibition by aztreonam/avibactam (fixed concentration of 4mg/L) at a minimum inhibitory concentration (MIC) of 0.25 mg/L but was resistant to ceftazidime/avibactam (MIC > 16 mg/L) and aztreonam alone (MIC > 16 mg/L) according to CLSI breakpoints.

**Conclusion:**

The *in vitro* activity of aztreonam and ceftazidime/avibactam against an NDM-5-producing *K. pneumoniae* was demonstrated with a modified superposition method. This may serve as a simplistic and readily available technique for rapidly determining antimicrobial activity in these difficult to treat pathogens.

**Disclosures:**

**Justin Schmetterer, PharmD, PhC, BCIDP**, AbbVie Speaker Bureau: Honoraria **Yao Lin, MS**, Abbvie Inc: employee|Abbvie Inc: Stocks/Bonds.

